# Supplementary material for: N-3 Polyunsaturated Fatty Acids and Their Lipid Mediators as A Potential Immune–Nutritional Intervention: A Molecular and Clinical View in Hepatic Disease and Other Non-Communicable Illnesses
Source: Nutrients. 2021 Sep 26;13(10):3384. doi: 10.3390/nu13103384 (PMC8539469; doi:10.3390/nu13103384)
Supplement: Supplementary file 1 [file nutrients-13-03384-s001.zip › nutrients-1361115-supplementary.pdf]

Supplementary tables

Table S1. NAFLD clinical trial

Nº	Clinical trial Code	Sponsor	Country	Results posted or published	Phase of study	Omega 3´composition
1	NCT02201160	St. Justine's Hospital	Canada	Not provided	in process	300 mg/day n-3 PUFA (EPA+DHA)
2	NCT02923804	Pronova BioPharma	USA	Not provided	completed	1 g n-3 PUFA (EPA+DHA)
3	NCT02647294	General University Hospital	Czech Republic	Not provided	completed	3,6 g/day n-3 PUFA (EPA+DHA) Maxicor
4	NCT03528707	Bogomolets National Medical University	Ukraine	Kobiliak et al (2018) [117]	completed	10 mg/day Symbiter Omega ®: (1-5%), supplemented with 14 alive probiotic strains: Lactobacillus + Lactococcus (6×1010 CFU/g), Bifidobacterium (1×101
5	NCT00230113	National Institute of Diabetes and Digestive and Kidney Diseases	USA	Not provided	completed	4 g fish oil per day (EPA+DHA)
6	NCT01277237	Edinburgh University	United Kingdom	Not provided	completed	4g/day Omacor ®
7	NCT00819338	Nottingham University	United Kingdom	Not provided	completed	5 g/day Efamax ®
8	NCT00760513	University Hospital Southampton NHS Foundation Trust	United Kingdom	Scorletti et al (2014) [118]; Scorletti et al (2014-2)[119]	completed	4g/day Omacor ®
9	NCT01936779	Oxford University	United Kingdom	Green et al (2020)[120]	completed	4 g/day n-3 PUFA (EPA+DHA)
10	NCT04281121	Ain Shams University	Egypyt	Not provided	completed	400 mg/day per 10 Kg of body weight (EPA+DHA)
11	NCT01056133	Canadian Institutes of Health Research (CIHR)/American College of Gastroenterology	Canada	Not provided	completed	2g/day n-3 PUFA (0.82/0.44 g of EPA/DHA)
12	NCT02354976	AstraZeneca	Sweden	Oscarsson et al (2018)[121]	completed	4 g/day n-3 PUFA (EPA+DHA)
13	NCT03132662	McMaster University	Canada	Lewis et al (2006)[122]; Capanni et al (2006)[123]; Spadaro et al (2008) [124]; Lanelli et al (2013) [125]	unknown	3g/day Oceano3 ® 1000 mg Krill Oil tabs (150 mg EPA + 90 mg DHA)
14	NCT04198805	Naga P. Chalasani	USA	Not provided	in process	1.89 g/day DHA EE and 1.86g/day DHA EE + 1g Vitamine E.
15	NCT01556113	Yale University	USA	Van Name et al (2020)[126]	completed	normocaloric low n-6:n-3 PUFA ratio
16	NCT01285362	Columbia University	USA	Not provided	completed	4g/day n-3 PUFA (465mg of EPA + 375 mg of DHA)
17	NCT00681408	Virginia University	USA	Argo et al (2015)[127]	completed	3 g/day n-3 PUFA (EPA+DHA)
18	NCT00694746	Boston University	USA	Not provided	Termined anticipated	4 g/day n-3 PUFA (EPA+DHA) (Omacor® or Lovaza®)
19	NCT02098317	Bambino Gesù Hospital and Research Institute	Italy	Della Corte et al (2016)[159]	completed	500 mg DHA plus Vitamin D 800 IU
20	NCT03380416	Federico II University	Italy	Della Pepa et al (2020)[128]	completed	2.6 g/day omega-3 (EPA+DHA)and 9.6g/day omega-6
21	NCT00845845	Illinois at Chicago University	USA	results at clinicaltrials.gov (no journal published)	completed	4 g/day n-3 PUFA (EPA+DHA) Lovaza®
22	NCT01992809	Sao Paulo General Hospital University	Brazil	Nogueira et al (2016)[162]	completed	3g/day n-3 PUFA (α linolenic acid/ 64%, eicosapentaenoic acid (EPA)/16% and docosahexaenoic acid (DHA)/21%)
23	NCT03801577	BASF AS	USA	Not provided	unknown	4g/day n-3 PUFA (EPA + DHA) Hepaxa®
24	NCT00323414	National Institute of Diabetes and Digestive and Kidney Diseases (NIDDK)	USA	Yonousi et al (2004)[129]; Dasarathy et al (2015)[130]	completed	6g/day n-3 PUFA (360 mg EPA and 240 mg DHA)
25	NCT02148471	Johane Allard / Canadian Liver Foundation	Canada	Da Silva (2014)[131]	completed	Observational: omega-3 dietary intakes
26	ChiCTR1800014419	Shangai Jiao Tong University	China	Not provided	in process	2g/day n-3 PUFA (EPA: DHA ratio of 150:500)
27	ChiCTRTRC12002380	Third Military Medical University	China	Qin et al (2015)[132]	Completed	4g/day n3 PUFA (182 mg of EPA + 129 mg of DHA)
28	U1111-1201-3408	Federal University do Rio de Janeiro	Brazil	Not provided	in process	3g/day n-3 PUFA (420 mg of EPA + 220 mg of DHA)
29	U1111-1229-8526	Federal University de Sergipe	Brazil	Not provided	in process	3g/day n-3 PUFA (EPA+DHA)
30	NCT01547910	Piotr Socha	Poland	JancZyk et al (2013)[157]	completed	1g/day n-3 PUFA (1300 mg EPA +450 mg DHA)

**31**	2012-001975-36	AZIENDA UNIVERSITARIA POLICLINICO UMBERTO I DI ROMA	Italy	Pacifico et al (2015)[158]	completed	250 mg/day DHA
**32**	CTRN12610000351011	Sidney University	Australia	Parker et al (2019)[160]	completed	2g/day n-3 PUFA 588 mg EPA and 412 mg DHA

Table S2. NASH clinical trial information

**№**	**Clinical trial Code**	**Sponsor**	**Country**	**Results posted or published**	**Phase of study**	**Omega 3´composition**
**1**	NCT01154985	Mochida Pharmaceutical Company, Ltd.	Japan	Sanyal et al (2014)[133]	completed	EPA-E 1800 mg/day or EPA-E 2700 mg/day
**2**	NCT01934777	Bambino Gesù Hospital and Research Institute	Italy	Not provided	completed	250 mg DHA plus Vitamin E (39 UI) plus 201 mg Choline
**3**	NCT03972319	Khoo Teck Puat Hospital	Singapore	Lanelli et al (2013)[125]	in process	2g/day n-3 PUFA (600mg Eicosapentaenoic acid (EPA), 1400mg Docosahexaenoic acid (DHA)
**4**	NCT02966899	Providence VA Medical Center	USA	Not provided	unknown	4g/day n-3 PUFA (EPA+DHA)
**5**	NCT02279407	AstraZeneca	Sweden	Eriksson et al (2018) [134]	completed	4g/day n-3 PUFA (EPA+DHA) + 10mg dapaglifozin
**6**	NCT03006016	Centre Hospitalier Universitaire de Nice	France	Not provided	unknown	1.6 g/day n-3 PUFA
**7**	UMIN000012886	Chiba University Hospital	Japan	Not provided	completed	4g/day n-3 PUFA (EPA+DHA)
**8**	2014-003637-26	AstraZeneca	Sweden	Not provided	completed	4g/day Epanova ®

Table S3. Liver parenteral nutrition clinical trial information

**№**	**Clinical trial Code**	**Sponsor**	**Country**	**Results posted or published**	**Phase of study**	**Omega 3´composition**
**1**	NCT02334293	Carle Foundation Hospital	USA	Not provided	unknown	Omegaven®
**2**	NCT02721277	University of Florida	USA	Results at clinicaltrials.gov (no journal published)	completed	3 g/kg/day SMOFlipid®
**3**	NCT02370251	University of Oklahoma	USA	Results at clinicaltrials.gov (no journal published)	completed	of 1 g/kg/day Omegaven®(parenteral nutrition)
**4**	NCT02412566	Baylor College of Medicine	USA	Not provided	in process	3 gm/kg/day (by continuous infusion) SMOFlipid®
**5**	NCT01284049	Assistance Publique - Hôpitaux de Paris	France	Not provided	completed	0.5 and 1 g/kg/infusion Omegaven 10%®
**6**	NCT00793195	The Hospital for Sick Children	Canada	Diamond et al (2018)[135]	unknown	SMOFlipid 20% (according the percentage of the subject's caloric intake consumed parenterally)
**7**	NCT03044639	Stanley Dudrick's Memorial Hospital	Poland	Klek et al (2018)[136]	completed	0.8-1.0 g/kg/day SMOFlipid®
**8**	NCT01323907	Sivan Kinberg	USA	Not provided	withdraw	0.5gram/kg/day Omegaven®

**9**	NCT02780193	Rush University Medical Center	USA	Gura et al (2008)[137];Puder et al (2009)[138]	completed	1 g/kg/day Omegaven®
**10**	NCT01845116	Atrium Health	USA	Gura et al (2008)[137];Puder et al (2009)[138]	completed	0.5g/kg/day Omegaven®
**11**	NCT01297933	Children's Hospital of Philadelphia	USA	Diamond et al (2018)[135]	completed	1 g/kg/day Omegaven®
**12**	NCT02121769	St. Luke's Health System, Boise, Idaho	USA	Not provided	completed	1 g/kg/day Omegaven®
**13**	NCT00738101	Baylor College of Medicine	USA	Gura et al (2021) [139]; Gura et al (2020) [140]; Premkumar et al (2013) [141]	completed	1 g/kg/day Omegaven®
**14**	NCT01565278	Johane Allard	Canada	Jurewitsch et al (2010)[142]; Fernandes et al (2011)[143]	completed	Intralipid+Omegaven: 0.25 g/kg/TPN Intralipid day+0.4 g/kg/TPN
**15**	NCT01191177	Boston Children's Hospital	USA	Not provided	withdraw	4g/day n-3 PUFA (Lovaza®) oral
**16**	NCT02534077	Mednax Center for Research, Education, Quality and Safety	USA	Not provided	completed	1 g/kg/day Omegaven®
**17**	NCT01194063	Kapiolani Medical Center For Women & Children	USA	Lee et al (2009)[144]; deMeijer (2010) [145]	completed	1 g/kg/day Omegaven®
**18**	NCT04697888	Children's Mercy Hospital Kansas City	USA	Results at clinicaltrials.gov (no journal published)	completed	1 g/kg/day Omegaven®
**19**	NCT00862446	Vanderbilt University Medical Center	USA	Results at clinicaltrials.gov (no journal published)	completed	1 g/kg/day Omegaven®
**21**	NCT01157780	University of Tennessee	USA	Not provided	unknown	1 g/kg/day Lovaza®
**22**	NCT00826020	University of Nebraska	USA	Mercer et al (2013)[146]	completed	1 g/kg/day Omegaven®

Table S4. HCC clinical trial information

Nº	Clinical trial Code	Sponsor	Country	Results posted or published	Phase of study	Omega 3´composition
**1**	NCT00168987	Charite University	Germany	Not provided	completed	100 ml EPA+DHA
**2**	NCT02321202	Huazhong University of Science and Technology	China	Zhang et al (2017)[88]	completed	Structolipid ® + 1 g/kg/day Omegaven®
**3**	NCT01819961	Wei Zhou	China	Not provided	unknown	Structural Fat Emulsion Injection 250ml and fish oil 100ml (Omegaven®)

Table S5. Healthy people clinical trial information

**Nº**	**Clinical trial Code**	**Sponsor**	**Country**	**Results posted or published**	**Phase of study**	**Omega 3´composition**
**1**	NCT01482689	University of Oslo	Norway	Holvik et al (2012)[147]	Completed	0.5 g n-3 PUFA (EPA+DHA)
**2**	NCT04609423	Oslo University Hospital	Norway	Not provided	In process	1.2 g of n-3 PUFA (DHA 0,6g and EPA 0,4g)

Table S6. HVC clinical trial information

**Nº**	**Clinical trial Code**	**Sponsor**	**Country**	**Results posted or published**	**Phase of study**	**Omega 3´composition**

1	NCT00547716	University of Missouri, Kansas City	USA	Not provided	Withdraw	4g/day Omacor@
2	NCT00408304	Bnai Zion Medical Center	Israel	Not provided	Unknown	not informed

Table S7. Mayor liver resection clinical trial information

Nº	Clinical trial Code	Sponsor	Country	Results posted or published	Phase of study	Omega 3´composition
1	NCT01884948	University of Zurich	Switzerland	Linecker et al (2015)[148]; Linecker et al (2020)[149]	Unknown	100 ml I.V Omegaven®
2	NCT01256047	Chiba University	Japan	Uno et al (2016)[150]	Completed	1L/day (5 days) Oral IMPACT ®

Table S8. Hepatic metastases clinical trial information

Nº	Clinical trial Code	Sponsor	Country	Results posted or published	Phase of study	Omega 3´composition
1	NCT03428477	Yorkshire Cancer Research	USA	Not provided	In process	4g/day EPA EE
2	NCT01070355	University of Leeds	United Kingdom	CockBain et al (2014)[151]	completed	2g/day EPA)
3	NCT04682665	University of Leeds	United Kingdom	Not provided	In process	4g/day EPA EE
4	NCT00942292	University Hospitals, Leicester	United Kingdom	Al-Taan et al (2013)[152]	completed	Lipidem- fish oil emulsion
5	NCT03428477	Yorkshire Cancer Research	USA	Not provided	In process	4g/day EPA EE

Table S9. Liver Transplant clinical trial information

Nº	Clinical trial Code	Sponsor	Country	Results posted or published	Phase of study	Omega 3´composition
1	NCT04030065	Institute of Liver and Biliary Sciences, India	India	Not provided	unknown	100 ml n-3 PUFA
2	NCT01957943	Mansoura University	Egypt	Not provided	unknown	SMOFlipid 20%
3	NCT02544919	Mansoura University	Egypt	Not provided	unknown	1 g/kg/day-1 SMOFlipid
